# Employing Xception convolutional neural network through high-precision MRI analysis for brain tumor diagnosis

**DOI:** 10.3389/fmed.2024.1487713

**Published:** 2024-11-08

**Authors:** R. Sathya, T. R. Mahesh, Surbhi Bhatia Khan, Areej A. Malibari, Fatima Asiri, Attique ur Rehman, Wajdan Al Malwi

**Affiliations:** ^1^Department of Computer Science and Engineering, SRM Institute of Science and Technology, Ramapuram, Chennai, India; ^2^Department of Computer Science and Engineering, JAIN (Deemed-to-be University), Bengaluru, India; ^3^School of Science, Engineering and Environment, University of Salford, Manchester, United Kingdom; ^4^Adjunct Research Faculty at the Centre for Research Impact and Outcome, Chitkara University, Chandigarh, Punjab, India; ^5^Department of Industrial and Systems Engineering, College of Engineering, Princess Nourah Bint Abdulrahman University, Riyadh, Saudi Arabia; ^6^College of Computer Science, Informatics and Computer Systems Department, King Khalid University, Abha, Saudi Arabia; ^7^Suleman Dawood School of Business, Lahore University of Management Sciences, Lahore, Pakistan

**Keywords:** brain tumor classification, medical imaging, deep learning, convolutional neural networks (CNN), Xception architecture, transfer learning

## Abstract

The classification of brain tumors from medical imaging is pivotal for accurate medical diagnosis but remains challenging due to the intricate morphologies of tumors and the precision required. Existing methodologies, including manual MRI evaluations and computer-assisted systems, primarily utilize conventional machine learning and pre-trained deep learning models. These systems often suffer from overfitting due to modest medical imaging datasets and exhibit limited generalizability on unseen data, alongside substantial computational demands that hinder real-time application. To enhance diagnostic accuracy and reliability, this research introduces an advanced model utilizing the Xception architecture, enriched with additional batch normalization and dropout layers to mitigate overfitting. This model is further refined by leveraging large-scale data through transfer learning and employing a customized dense layer setup tailored to effectively distinguish between meningioma, glioma, and pituitary tumor categories. This hybrid method not only capitalizes on the strengths of pre-trained network features but also adapts specific training to a targeted dataset, thereby improving the generalization capacity of the model across different imaging conditions. Demonstrating an important improvement in diagnostic performance, the proposed model achieves a classification accuracy of 98.039% on the test dataset, with precision and recall rates above 96% for all categories. These results underscore the possibility of the model as a reliable diagnostic tool in clinical settings, significantly surpassing existing diagnostic protocols for brain tumors.

## 1 Introduction

Aberrant cell development inside the brain or central spinal canal is called a brain tumor. Depending on their growth rate and location, brain tumors can disrupt normal brain function and are potentially life-threatening. Brain tumors are typically categorized into primary tumors, which begin in the brain, and secondary or metastatic tumors, which spread from other regions of the body (1).

**Figure 1:**
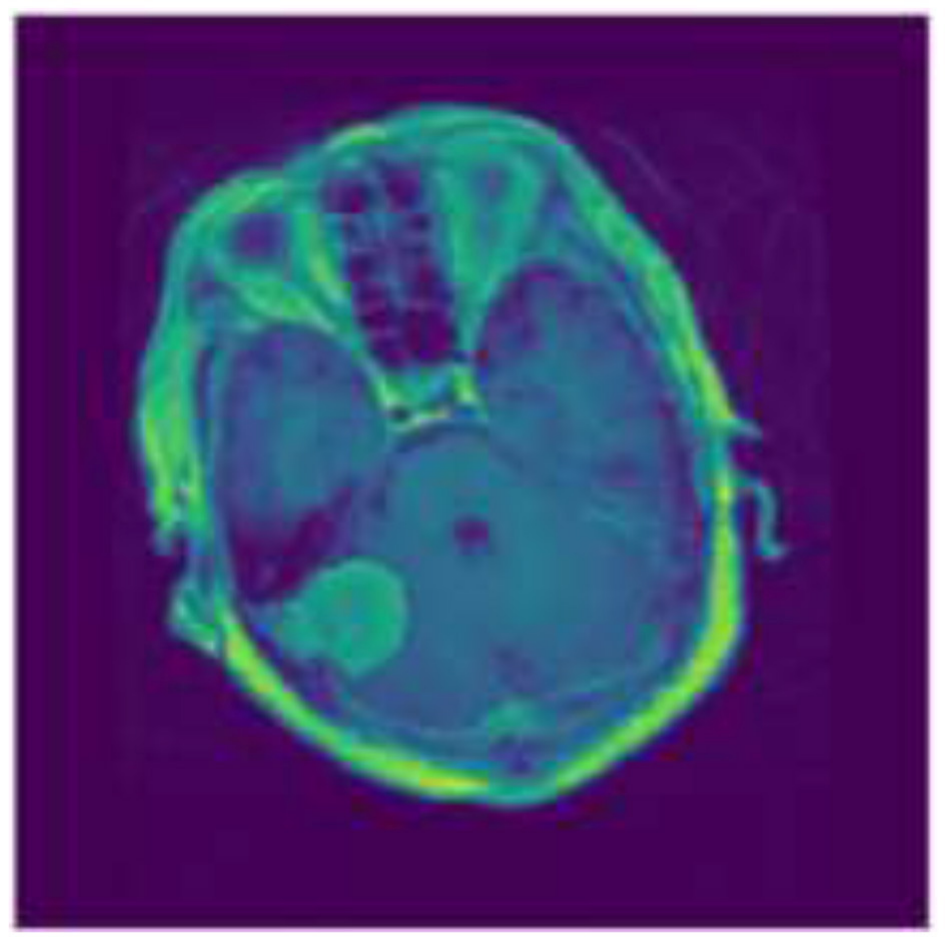
Meningiomas tumor.

Tumors that Originating from the meninges, which are the protective membranous layers enveloping the brain and spinal cord, are called meningiomas. Typically benign, meningiomas are generally amenable to surgical interventions, with a favorable prognosis following complete resection. However, their location and size can cause significant neurological impairments if they impinge on critical areas of the brain or spinal structures (2). Figure 1 shows the sample image for Meningiomas tumor.

**Figure 2:**
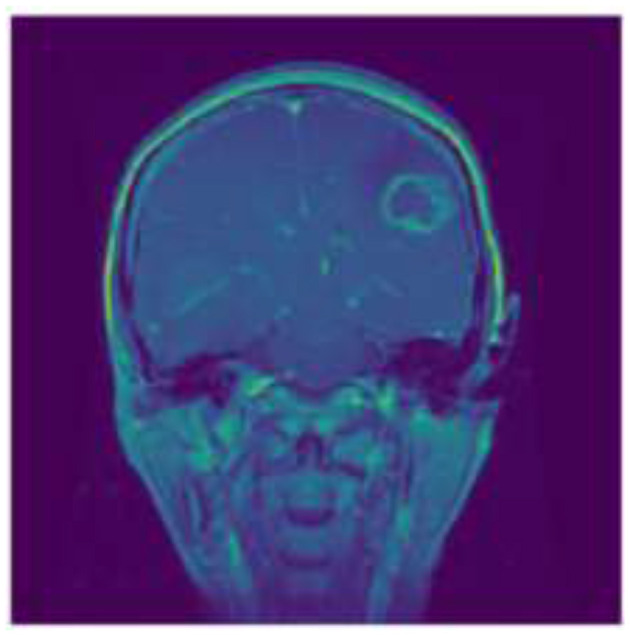
Gliomas tumor.

Gliomas, which originate from the glial cells that support nerve cells in the brain, represent a more heterogeneous group characterized by varying degrees of malignancy. The origin of these tumors cells determines the category in which they fall, such as astrocytoma's or oligodendrogliomas. High-grade gliomas (grades III and IV), such as glioblastoma multiforme, are noted for their aggressive nature and poor prognosis, often infiltrating surrounding brain tissue to an extent that makes complete surgical removal challenging (3). Figure 2 shows the sample image of Gliomas Tumor.

**Figure 3:**
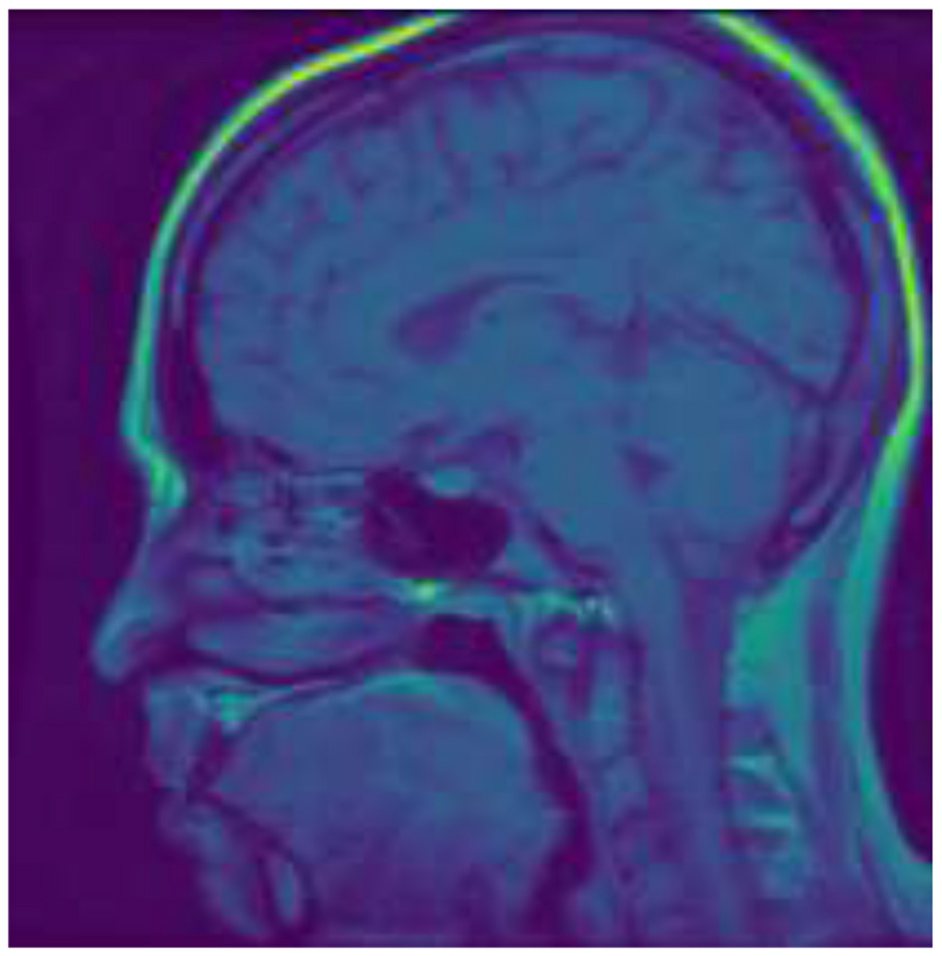
Pituitary tumor.

Pituitary tumors arise from the pituitary gland, a critical hormone-regulating organ seated at the base of the brain. While these tumors are predominantly benign, they can significantly affect bodily functions due to hormonal imbalances they induce, manifesting symptoms such as vision disturbances, infertility, and other endocrine disorders. The treatment protocol may involve surgery, medication, or radiation therapy, depending on the tumor's size, growth rate, and hormonal activity (4). Figure 3 shows the sample image of Pituitary tumor.

These modalities allow for detailed visualization of tumor size, location, and potential infiltration into adjacent tissues, thereby guiding therapeutic strategies. For instance, employing machine learning models in image processing can improve both the accuracy and speed of tumor classification, aiding radiologists in making well-informed diagnostic and treatment decisions.

The motivation behind this study's requirement to improve the precision and effectiveness of brain tumor diagnosis using methods for medical imaging such as MRI. Traditional methods in medical imaging, whose interpretations can vary significantly complex nature of brain imaging. Furthermore, existing automated methods often struggle with issues like overfitting, generalization, and computational inefficiency, making them less effective in clinical settings. The advent of deep learning offers a promising avenue to overcome these challenges, given its prowess in pattern recognition and feature extraction in complex datasets. However, the deployment of such models in a medical context requires careful adaptation and validation to meet the stringent accuracy and reliability standards necessary for clinical diagnosis (5, 6).

The objectives of the research:

- Develop a robust model that can precisely classify brain tumors into meningioma, glioma, or pituitary tumor.
- Implement advanced techniques such as batch normalization and dropout help reduce overfitting and enhance the generalization capacity of the model to new, unseen data.
- Provide a framework for further adaptation and use of deep learning models in the medical imaging field, promoting more accurate and rapid diagnosis processes.

The further organization of the paper is as follows: Section 2 presents the state-of art existing methodologies, Section 3 depicts the workflow of the proposed model. Section 4 presents the results of the proposed model, Section 5 presents the comparison of proposed model with the state-of-art methodologies. Finally, Section 6 concludes with the justification of the results.

## 2 Related work

A variety of studies have explored different aspects of applying deep learning models to enhance the diagnosis and classification of brain tumors. These studies have generally focused on enhancing the precision, efficiency, generalizability predictive models. Despite these advancements, several challenges remain.

Traditional methods for brain tumor analysis primarily involved manual inspection of medical images by experienced radiologists. These techniques depended on visual assessments using MRI, CT scans, and other imaging modalities to identify irregularities suggestive of tumors. The accuracy of these diagnoses heavily relied on the individual expertise and experience of the medical professionals, leading to variability in diagnostic accuracy and potential for human error (7). Additionally, these methods were time-consuming and often required corroborative tests to confirm diagnoses.

With the advent of digital imaging and increased computational power, early machine learning techniques began to be integrated into the analysis of brain tumors. Earlier machine learning models tried to automate the process of extracting features from photos and determining important attributes that would indicate the existence of a tumor. However, these methods still struggled with handling the high dimensionality of medical images and often required extensive preprocessing of data to be effective. They provided a foundation for automated analysis but were limited by the quality and amount of the data available, which could introduce biases (8).

Convolutional Neural Networks (CNNs) became particularly influential because of their capacity to automatically and effectively learn features from raw imaging data. Unlike traditional machine learning techniques, CNNs could handle complex image data directly, learning hierarchical features that improved classification and segmentation tasks (9). Initial deep learning models, such as AlexNet and later more complex architectures like GoogLeNet and VGG, demonstrated substantial improvements in accuracy and efficiency, reducing reliance on manual feature engineering and significantly improving generalization across diverse datasets.

Recent advances in deep learning have focused on enhancing the precision, efficiency, and interpretability of models for brain tumor analysis. Innovations like transfer learning enable the use of pre-trained networks on extensive datasets, which can subsequently be fine-tuned for specific medical imaging tasks, such as brain tumor categorization. This approach has dramatically reduced the need for large domain-specific datasets, which are hard to come by in medical fields. Furthermore, newer architectures like the Xception model, which incorporates depthwise separable convolutions, offer improved performance by increasing the model's ability to learn from medical images while being computationally efficient. (6). Table 1 Shows a summary of studies with their specific methods and accuracy for brain tumor classification.

**Table 1:** Literature review of existing models.

**References**	**Accuracy**	**Remark**
Pedada et al. (10)	93.40% and 92.20%	This study presents a novel approach with significant accuracy improvements, demonstrating a strong contribution to automated brain tumor segmentation.
Saeedi et al. (11)	96.47 %	The study presents a comprehensive and innovative approach, combining deep learning techniques, in order to greatly improve the accuracy of early-stage brain tumor identification and categorization.
Wozniak et al. (12)	96%	The study presents a unique CLM model that effectively enhances the efficiency and precision of CT brain scan evaluations, highlighting its potential for future advancements in medical imaging.
Gayathri et al. (13)	94%	The study successfully illustrates the VGG-16 model's potential for precise brain tumor identification, achieving strong performance metrics and outperforming some existing methods, though it highlights areas for further improvement and research.
Hossain et al. (14)	96.97%	This research proposed MBINet for brain tumor classification.
Mohamed et al. (15)	95.44%	The work emphasizes the usefulness of CNNs in improving brain tumor identification.
Thillaikkarasi and Saravanan (16)	84%	Create an automated and exact brain tumor segmentation system using a kernel-based Convolutional Neural Network (CNN) and Multiple Support Vector Machines (M-SVM) in MRI images.
Senan et al. (17)	95.10%	Alexnet + SVM
Gayathri et al. (13)	94%	Evaluate how well the VGG-16 architecture performs when using deep learning to reliably identify brain tumors.
Haq et al. (18)	91.28%	The hybrid model was developed using an ensemble approach.

The summary of existing methods in brain tumor analysis range from traditional methods, which suffer from subjectivity, variability in diagnostic accuracy, and time-consuming processes, to early machine learning techniques that struggle with the high dimensionality of medical images and require extensive data preprocessing and manual feature selection, introducing potential biases. Deep learning, while improving accuracy and efficiency, demands significant computational resources and often lacks interpretability, crucial in medical applications. Recent advances, including transfer learning and integrative approaches like federated learning, still face challenges such as dependency on large datasets, difficulties in generalization across diverse imaging equipment, complex integration requirements, and privacy concerns in data sharing. These difficulties show how much more study is required to improve these technologies for better adaptability and understanding in clinical settings.

## 3 Methodology

**Figure 4:**
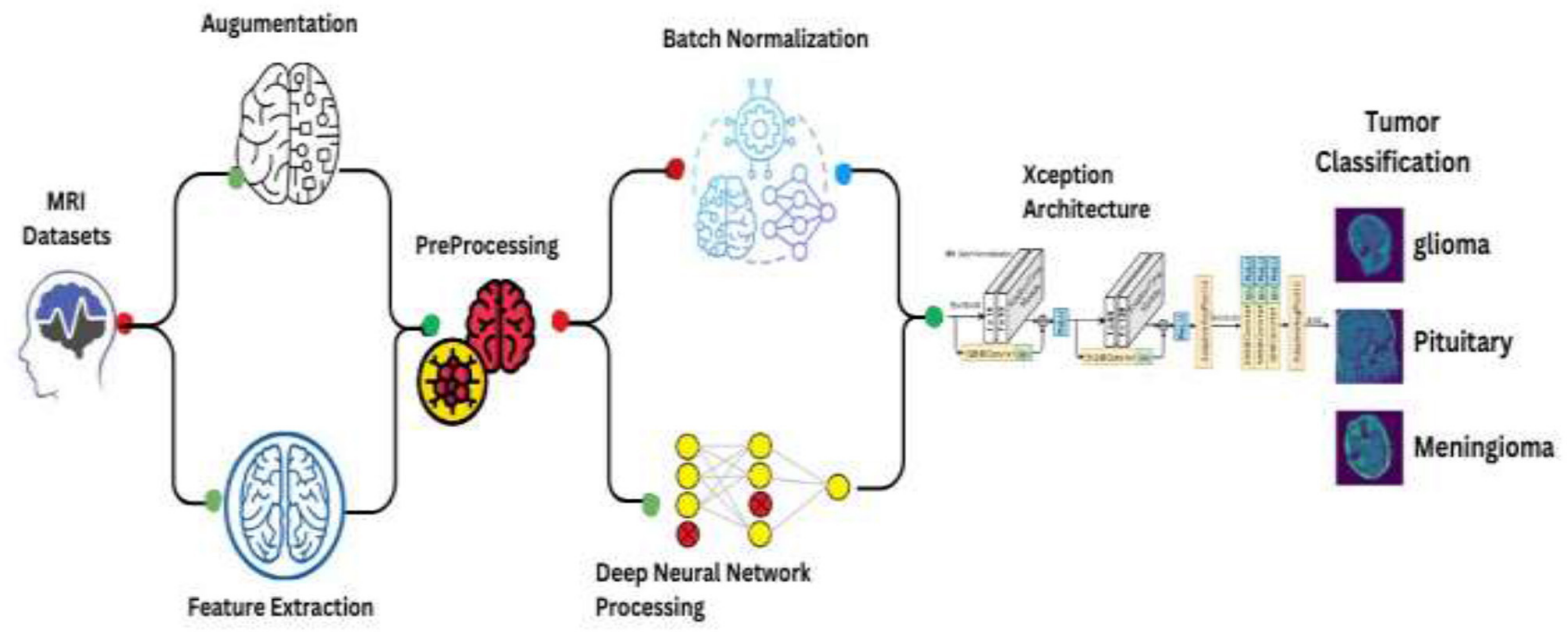
Architectural visualization of the proposed model.

This comprehensive approach integrates data preprocessing, augmentation, and the deployment of a convolutional neural network (CNN) leveraging the Xception architecture, followed by statistical analysis. Figure 4 depicts Schematic of the enhanced Xception CNN architecture tailored for brain tumor classification.

### 3.1 Dataset description

The MRI images utilized in this study are derived from a publicly accessible dataset created and shared by Jun Cheng. The dataset used in this study consists of MRI images, which are categorized into three main types: meningioma, glioma, and pituitary tumors. The images are originally grouped into respective folders by class, facilitating straightforward extraction and manipulation. The dataset is substantial enough to train a deep learning model, with each category containing hundreds of images, thereby enabling diverse tumor features. The variability within each category includes different stages and sizes of tumors, further enriching the dataset's complexity and providing a robust challenge for the deep learning model to tackle, ensuring it learns to identify subtle and critical differences among the tumor types (19). The dataset comprises T1-weighted contrast-enhanced images from 233 patients. These images were initially presented in '.mat' format and were converted to “.png” format for this study. The selection criteria for the images included in the dataset were primarily based on the clarity and diagnostic relevance of the MRI scans, ensuring that each image distinctly represented the tumor characteristics necessary for effective training of the deep learning model. Images that did not meet these quality standards were excluded to maintain the integrity and reliability of the dataset.

**Figure 5:**
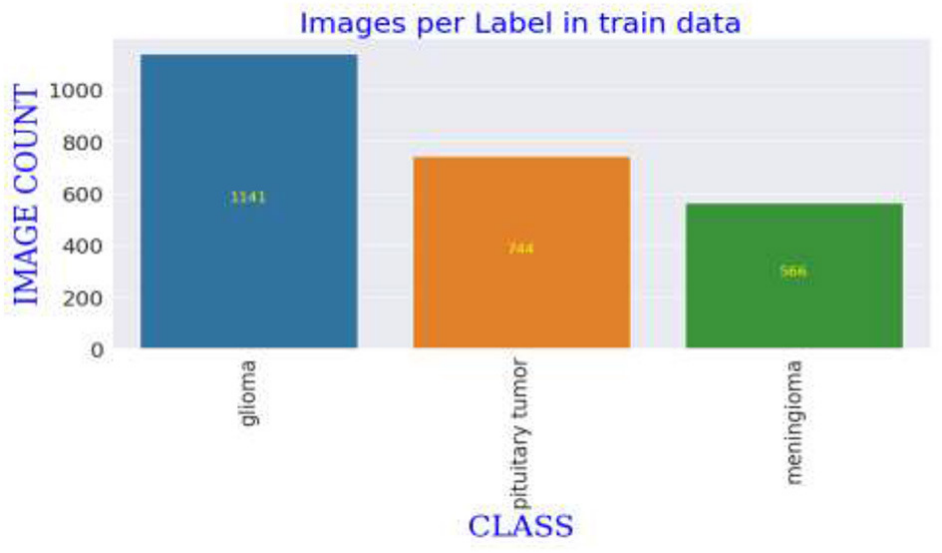
Distribution of MRI scan images across tumor types in training dataset.

In this study, potential biases related to patient demographics such as age, gender, and ethnicity, which may impact MRI characteristics of brain tumors, are acknowledged and addressed to develop a fair diagnostic tool. To mitigate these biases, a diverse dataset including images from a broad demographic spectrum is compiled, and data augmentation techniques like rotation, scaling, and flipping are utilized to simulate varied tumor appearances. The model undergoes stratified cross-validation to ensure consistent accuracy across different demographic groups. Algorithms specifically designed to detect and correct biases assess the model's performance to prevent disparities in diagnostic accuracy. Continuous monitoring and regular updates in clinical settings further ensure the model adapts to new data, maintaining reliability and fairness across all patient groups, thereby enhancing its clinical applicability and trustworthiness. Figure 5 Displays representative MRI scans of each tumor type to demonstrate the input data quality and variety.

### 3.2 Data preprocessing

The preprocessing steps, including Data augmentation, image resizing and normalization, made a substantial contribution to the model's performance by guaranteeing dataset homogeneity and improving the model's capacity to generalize over a range of MRI images. Data preprocessing for MRI images is crucial for ensuring homogeneity and optimization for deep learning. Each image undergoes normalization, scaling pixel values from 0 to 1 to enhance the model's numerical stability and speed up convergence during training. Additionally, to accommodate the Xception architecture in this study, all images are resized to a uniform dimension of 224 × 224 pixels, eliminating size variability that could impact learning efficiency. Furthermore, data augmentation techniques such as random horizontal flipping and rotations are applied to the training dataset. These techniques mimic potential variations in clinical image capture, enhancing the model's generalizability and diagnostic accuracy in real-world settings.

**Figure 6:**
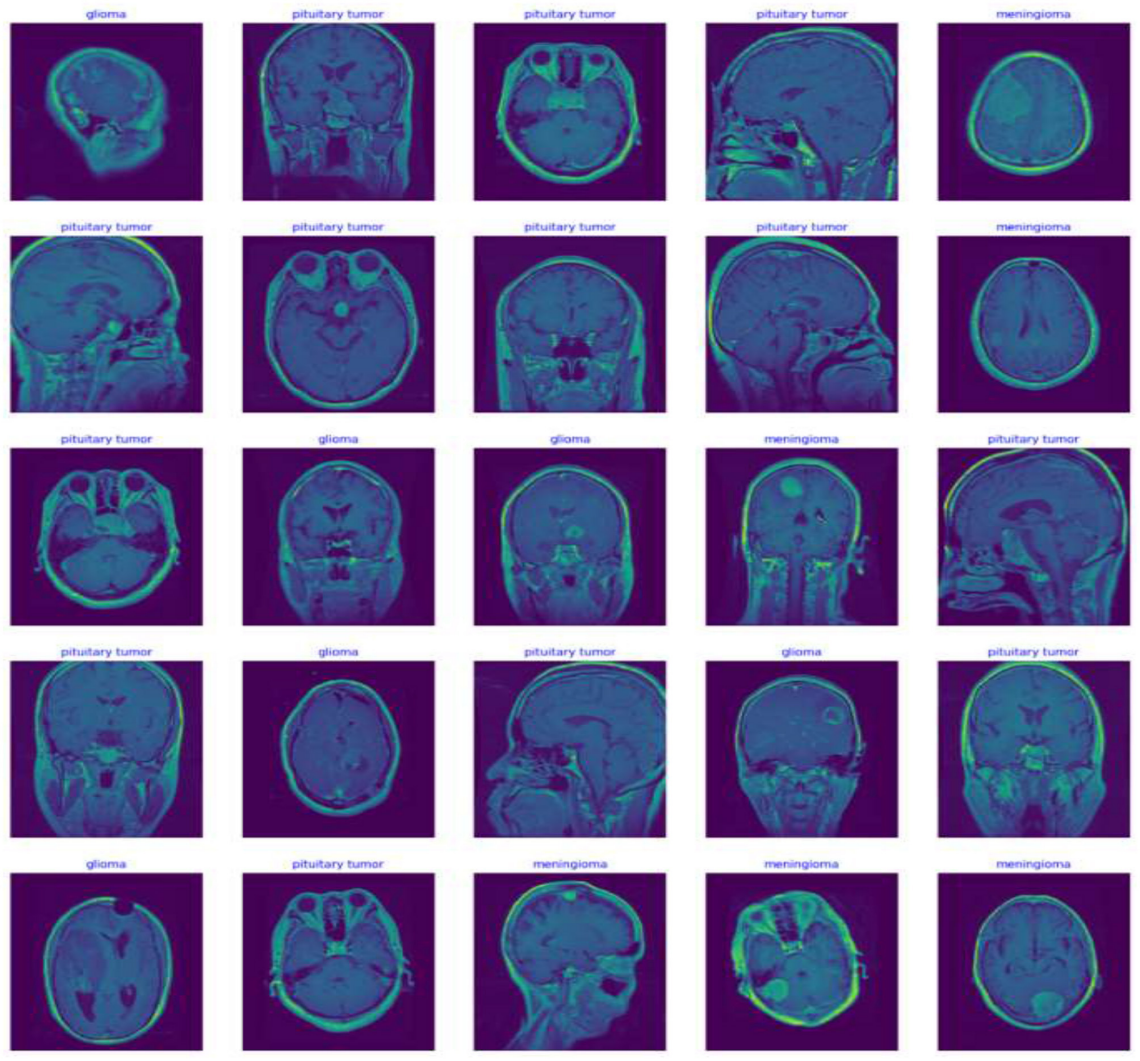
Sample images from the datasets.

Data augmentation simulates a variety of plausible scenarios that could occur during image capture. This process is particularly beneficial in medical imaging, where different patient positions or imaging angles can vary significantly. For instance, a brain tumor might appear in different locations and orientations depending on how the scan was conducted. augmentation techniques such as scaling, translation, and shear can mimic variations due to different scanner settings or patient movements (20). These transformations make the model more robust, enabling it to maintain high performance regardless of these variabilities in new, unseen images. Equation 1 represents the flipping transformation on an image. Equation 2 represents the rotation of an image by an angle θ, using the rotation matrix *R*(θ). Figure 6 shows the Examples of image transformations applied during data augmentation to enhance model robustness. Table 2 shows the detailed specific augmentation methods used, such as rotations and flips, to train the model.


(1) $$\begin{array}{l} I_{flipped}\left(x,y\right)=I\left(W-x,y\right) \end{array}$$

(2) $$\begin{array}{l} I_{rotated}\left(\theta \right)=R\left(\theta \right)\cdot I \end{array}$$

**Table 2:** Augmentation technique.

**Augmentation technique**	**Description**
Horizontal flip	Mirrors the image horizontally
Random rotation	Rotates the image by a random angle

#### 3.2.1 Normalization

It is applied to all images by scaling the pixel values to a range of 0 to 1, a crucial step for enhancing the model's training efficiency. This normalization not only aids in speeding up the convergence but also helps in maintaining numerical stability, which is essential for optimizing the gradient descent process. By guaranteeing that the scales of all input characteristics, in this case the pixel intensities, are comparable, the normalization process reduces the likelihood of encountering vanishing or exploding gradients, thereby facilitating a smoother and more stable learning trajectory (21). This step is vital for deep learning models, especially those dealing with high-dimensional data like images. Equation 3 normalizes pixel values *x* by dividing by 255 to scale between 0 and 1. Equation 4 updates the moving average of means during batch normalization, blending the previous average with the current batch's mean. Equation 5 updates the moving variance during batch normalization, combining the old variance with the variance from the current batch. Equation 6 explains adjustment to the normalized data using learned parameters γ and β in batch normalization.


(3) $$\begin{array}{l} x_{\text{norm}}=\frac{x}{255}\\ \mu_{\text{new}}=\text{momentum}\times \mu_{\text{old}}+\left(1-\text{momentum}\right) \end{array}$$

(4) $$\begin{array}{l} \;\times \text{sample\_mean} \end{array}$$

(5) $$\begin{array}{l} \sigma_{\text{new}}^{2}=\text{momentum}\times \sigma_{\text{old}}^{2}+\left(1-\text{momentum}\right)\\ \;\times \text{sample\_variance} \end{array}$$

(6) $$\begin{array}{l} \;\gamma x_{\text{norm}}+ \end{array}$$

#### 3.2.2 Image resizing

This uniformity is crucial as it standardizes the input size for the neural network, ensuring that the network architecture does not have to cope with variability in image size which could lead to inefficiencies in learning and performance. Resizing is typically performed before any augmentation or further processing of the original medical images. Incorporating dropout and batch normalization into the neural network architecture significantly enhances training and overall performance. Dropout, a regularization method, randomly ignores subsets of neurons during training, preventing the model from relying excessively on specific neurons or groups, thereby mitigating overfitting and encouraging the network to develop more robust features. Batch normalization tackles internal covariate shift by normalizing network activations, which stabilizes the training process and allows for higher learning rates, speeding up training and reducing overfitting. These techniques are vital in our network configuration, with dropout simulating the training of multiple networks by approximating different neuron subsets, enhancing generalization. Meanwhile, batch normalization facilitates more independent learning across layers, aids in maintaining effective gradient flow during backpropagation, and helps avoid the vanishing or exploding gradients often problematic in deep networks, ensuring more stable and efficient learning.

### 3.3 Deep learning techniques

The top layers of this deep learning model are removed to accommodate the integration of custom layers specifically designed for brain tumor classification. Following the feature extraction capabilities of Xception model, architecture is enhanced with a batch normalization layer that stabilizes the learning process, essential for adapting the model to the specific challenges of medical imaging. Subsequently, a dense layer comprising 256 neurons is incorporated. This layer utilizes both L2 and L1 regularization techniques to mitigate the risk of the model's overfitting, a crucial factor to take into inherent complexity and the finite nature of the dataset available for brain tumor studies. The strategic choice of the Xception model and the meticulous configuration of the subsequent layers exemplify a deliberate approach to leverage advanced machine learning techniques for enhanced diagnostic accuracy in medical imaging.

Transfer learning is pivotal in this research, utilizing a pre-trained Xception model originally developed on a diverse dataset of natural images to enhance brain MRI image analysis, specifically for tumor classification. By beginning with weights that have learned generic features, the model is fine-tuned with brain MRI images, allowing it to adapt these universal features to the specialized task of identifying tumors, thereby enhancing its generalizability across domains. This adaptability is particularly vital in medical settings, where the model must perform consistently across images from various MRI technologies that differ in calibration, imaging techniques, and might produce variations in image contrast, resolution, or anatomical positioning. Transfer learning obviates the need for extensive datasets typically required for training models from scratch—a significant advantage in medical imaging where obtaining large, annotated datasets can be challenging. By leveraging a model pre-trained on heterogeneous data, transfer learning not only helps prevent overfitting but also ensures that the model doesn't merely memorize specific training data but genuinely learns to discern underlying patterns indicative of tumors across different imaging conditions, thereby bolstering diagnostic accuracy and robustness (22). CNNs are particularly effective at capturing spatial hierarchies in data. Equation 9 adds an L2 penalty to the cost function to prevent overfitting by penalizing large weights. Equation 10 adds an L1 penalty to encourage sparsity in the neural network parameters. Equation 11 represents General form of the loss function including regularization term *R*(θ). Equation 12 explains the sigmoid activation function, used to map values to a (0, 1) range, typically in the output layer of binary classifiers. Equation 13 represents the output of a neural network layer.


(7) $$\begin{array}{l} J_{reg}\left(\theta \right)=J\left(\theta \right)+\lambda |\theta {|}^{2} \end{array}$$

(8) $$\begin{array}{l} J_{reg}\left(\theta \right)=J\left(\theta \right)+\lambda |\theta {|}_{1} \end{array}$$

(9) $$\begin{array}{l} J\left(\theta \right)=L\left(\theta \right)+\lambda R\left(\theta \right) \end{array}$$

(10) $$\begin{array}{l} \sigma \left(x\right)=\frac{1}{1+e^{-x}} \end{array}$$

(11) $$\begin{array}{l} y=f\left(Wx+b\right) \end{array}$$

Algorithm 1 encapsulate a thorough approach to classifying brain tumors using advanced deep learning techniques, tailored to specific requirements and challenges of medical image analysis.

**Algorithm 1:**
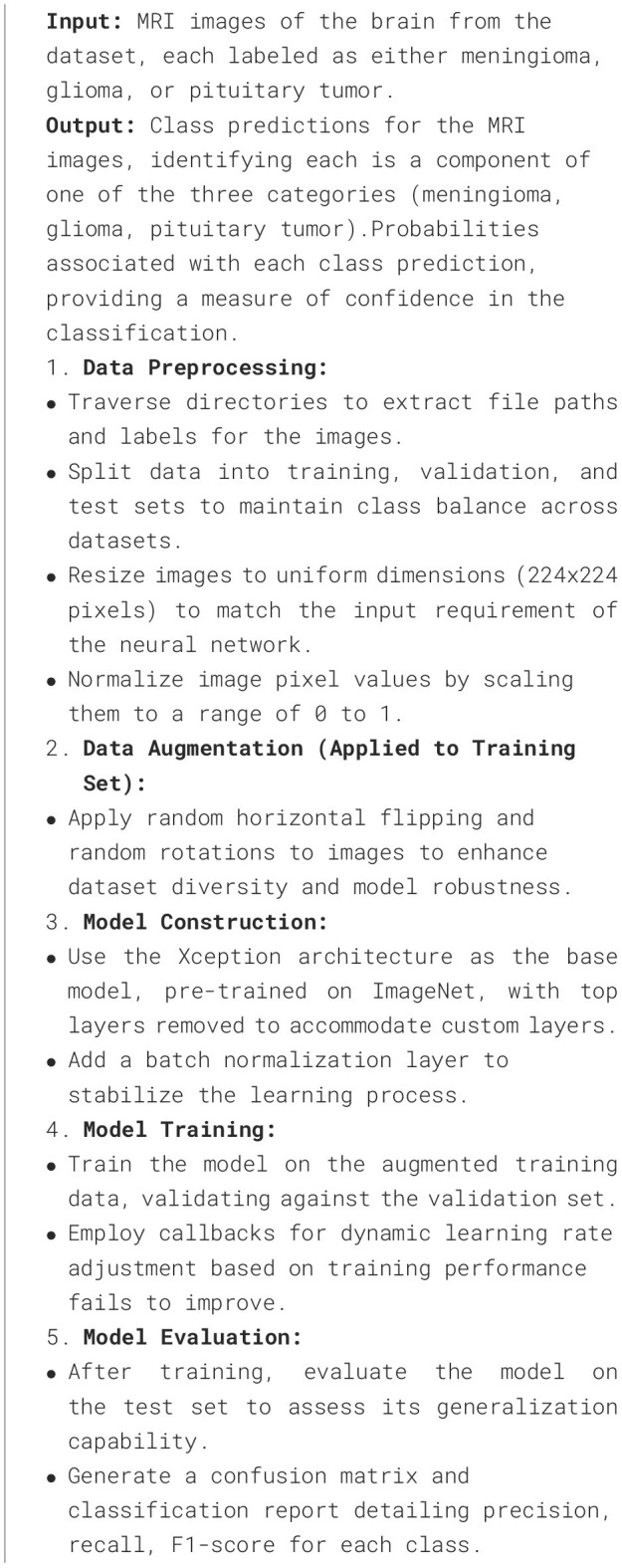
Brain tumor classification using deep learning.

The final layer of the deep learning model is configured as a softmax layer, which plays a critical role in the multi-class classification of the MRI images into three distinct categories of brain tumors meningioma, glioma, and pituitary tumor. This layer is essential for transforming the logits—outputs of the last neural network layer before the softmax—into probabilities by mapping the unbounded scores into a (0, 1) range that sums to one, effectively providing a probabilistic interpretation of the model's predictions. (Equation 14) depicts the softmax function used for multi-class classification, converting logits *z* into probabilities.


(12) $$\begin{array}{l} \sigma {\left(z\right)}_{i}=\frac{e^{z_{i}}}{\overset{K}{\underset{j=1}{\sum }}e^{z_{j}}} \end{array}$$

(13) $$\begin{array}{l} f\left(x\right)=\max\left(0,x\right) \end{array}$$

This model is compiled using the Adamax optimizer, a variant of the Adam optimizer that adapodes the learning rates determined by a gradient update moving window, rather than adding together all previous gradients (23). This makes it suitable for datasets with noisy gradients. The model undergoes training over several epochs with real-time monitoring.

The decision to employ the Xception model as the foundation of our deep learning framework was driven by several key considerations. Firstly, the Xception architecture is renowned for its depthwise separable convolutions, which allow it to perform more efficiently on less data while maintaining high accuracy. This characteristic is particularly advantageous given the complex nature of MRI brain tumor images, where subtleties in tumor morphology must be accurately captured with limited training examples. Xception's architecture is structured to enhance feature extraction through its use of channel-wise convolutions. This enables the model to learn more robust and discriminative features from medical images, a critical aspect when dealing with the high variability present in brain tumor appearances. The architecture also benefits from reduced computational demand compared to other complex models like InceptionV3 or ResNet, making it more suitable for applications where real-time processing is crucial. The Xception model has shown superior performance in previous benchmarks on image classification tasks, particularly those involving medical imaging data. Its ability to generalize well from training data to unseen data makes it an ideal choice for this study, aiming to improve diagnostic accuracy in the clinical evaluation of brain tumors. These factors collectively motivated the choice of the Xception model, ensuring that the architecture aligns well with the specific challenges and requirements of classifying brain tumors from MRI scans. The architecture of our deep learning model features a custom dense layer setup crucial for the final classification of brain tumors, incorporating a dense layer of 256 neurons to strike a balance between computational efficiency and the capability to capture complex patterns essential for distinguishing different brain tumor types. The choice of 256 neurons optimizes the learning of detailed features without imposing excessive computational demands or risking overfitting. For activation functions, the model employs the ReLU (Rectified Linear Unit) across these dense layers due to its efficacy in mitigating the vanishing gradient problem, which is prevalent in deep neural networks, and its capacity to introduce necessary non-linearity without compromising generalization. The final layer utilizes a softmax activation function, converting network outputs into a probabilistic distribution suitable for multi-class classification tasks such as tumor type identification. This setup ensures that softmax highlights the most likely class for each input, facilitating clear and interpretable predictions from the model.

### 3.4 Computational demands and optimization strategies

The Xception-based model, with its deep architecture and multiple layers, is computationally intensive, especially when handling high-resolution MRI images. Key performance metrics include FLOPS and memory usage. To adapt this model for real-time use, several optimizations can be implemented, model quantization reduces operation precision to speed up processing; pruning eliminates non-essential elements to simplify the network; and hardware accelerations like GPUs enhance processing speeds. Additional software strategies like efficient batch processing and parallel computing also boost performance. These adjustments lower computational demands and improve the model's responsiveness, essential for clinical settings where rapid diagnosis is critical.

To scale up for larger datasets and real-time data in clinical settings, distributed computing via Apache Spark or TensorFlow Distributed can parallelize MRI data processing, enhancing speed. Cloud platforms like Google Cloud or AWS provide scalable resources to match computational demands. For real-time streaming, optimizing the model architecture for low-latency, using asynchronous loading, and employing techniques like quantization and pruning are crucial. Edge computing places processing closer to data sources, minimizing delays and boosting responsiveness. Strategies like online learning ensure the model adapts continually to new clinical data, maintaining accuracy and relevance.

### 3.5 Statistical analysis

Following the model's training, it is tested against the unseen test set. It is used to analyze the model's predictive accuracy across the different classes, providing insights into any systematic errors in prediction (24). Equation 16 counts the number of instances where the true label is *i* and the predicted label is *j*, for constructing a confusion matrix. Table 3 depicts the number of true positive and false positive rates for each class, evaluating model classification accuracy.


(14) $$\begin{array}{l} C_{i,j}=\overset{N}{\underset{k=1}{\sum }}1\left(y_{k}=i\wedge \hat{y_{k}}=j\right) \end{array}$$

**Table 3:** Confusion matrix.

**True Label**	**Meningioma**	**Glioma**	**Pituitary**
Meningioma	150	10	5
Glioma	15	145	2
Pituitary Tumor	0	5	195

To compare the true positive rate to the false positive rate at different threshold values, ROC curves are plotted for each class. The AUC offers a single scalar value that represents the model's overall effectiveness in differentiating between classes.


(15) $$\begin{array}{l} TPR=\frac{TP}{TP+FN},FPR=\frac{FP}{TN+FP} \end{array}$$

(16) $$\begin{array}{l} AUC=\int_{0}^{1}TPR\left(FPR\right)d\left(FPR\right) \end{array}$$

### 3.6 Different metrics

Loss (Categorical Cross-Entropy) provides a quantitative measure of the model's prediction errors, indicating how well the probability distributions predicted by the model match the actual distribution of the labels.


(17) $$\begin{array}{l} L=-\overset{M}{\underset{i=1}{\sum }}y_{i}\log\left(\hat{y_{i}}\right) \end{array}$$

These class-specific metrics are critical for medical applications. Precision is important to minimize false positives. Recal ensures that the model detects as many positives as possible (25). Equations 20–22 measure precision (accuracy of positive predictions), recall (coverage of actual positives), and F1 score (balance between precision and recall), respectively (26).


(18) $$\begin{array}{l} \text{Precision}=\frac{\text{TP}}{\text{TP }+\text{ FP}} \end{array}$$

(19) $$\begin{array}{l} \text{Recall}=\frac{\text{TP}}{\text{TP }+\text{ FN}} \end{array}$$

(20) $$\begin{array}{l} \text{F}1=2\times \frac{\text{Precision }\times \text{ Recall}}{\text{Precision }+\text{ Recall}} \end{array}$$

MSE is calculated by squaring the differences between predicted and actual values, summing all these squared distinctions, and dividing the result. It emphasizes larger errors by squaring them, thus penalizing prediction errors disproportionately.


(21) $$\begin{array}{l} \text{MSE}=\frac{1}{\text{n}}\overset{\text{n}}{\underset{i=1}{\sum }}{\left({\text{y}}_{\text{i}}-\hat{{\text{y}}_{\text{i}}}\right)}^{2} \end{array}$$

The RMSE is derived by taking the square root of MSE, which quantifies the magnitude of prediction errors. This transformation aligns the error metrics with the original data's unit, enhancing interpretability by indicating the average distance between predicted and actual values. Equation 22 represents calling it back to the original units of the data and providing a clear measure of the average error magnitude.


(22) $$\begin{array}{l} \text{RMSE}=\sqrt{\frac{1}{\text{n}}\overset{\text{n}}{\underset{i=1}{\sum }}{\left({\text{y}}_{\text{i}}-\hat{{\text{y}}_{\text{i}}}\right)}^{2}} \end{array}$$

MAE calculates the average magnitude of prediction errors, disregarding their direction by treating all errors as positive values.


(23) $$\begin{array}{l} \text{MAE}=\frac{1}{\text{n}}\overset{\text{n}}{\underset{i=1}{\sum }}|{\text{y}}_{\text{i}}-\hat{{\text{y}}_{\text{i}}}| \end{array}$$

The model's robustness and the reliability of its performance metrics are assessed using k-fold cross-validation. This process involves dividing the entire dataset into k distinct subsets. The model is then trained on k-1 of these subsets, with the remaining subset used as the test set. This process is repeated k times, with each of the k subsets used exactly once as the test set. This method ensures that the model's performance is tested comprehensively across all available data, reducing variability and providing a more accurate estimate of its effectiveness.

To estimate the confidence intervals for the model's accuracy, the bootstrapping technique is employed. This involves repeatedly sampling with replacement from the dataset and training the model on each sample. The variance observed in the accuracy across these samples provides an estimate of the confidence interval. This statistical approach helps in understanding the stability of the model's predictions and provides a quantifiable measure of the uncertainty associated with the model's accuracy metrics.

These methods enhance the statistical rigor of the study by providing a clearer view of how the model might perform in real-world settings, where data may not always be as homogeneous as in controlled experiments. The addition of these statistical evaluations will help in substantiating the reliability of the model and its readiness for clinical application.

## 4 Experimentation and results

This study employed a sophisticated deep learning methodology, utilizing a convolutional neural network (CNN) specifically designed around the Xception architecture, to effectively classify brain tumors from a set of medical imaging data. Data augmentation methods included horizontal flipping of images, which is critical in diversifying the training dataset and enhancing model robustness (27). Additionally, normalization of pixel values was performed to ensure uniformity in the input data, which is essential for achieving consistent performance across various imaging conditions. These strategies were crucial in simulating a real-world environment for medical imaging diagnostics, thus providing a comprehensive test of the model's capabilities in accurately identifying and categorizing different brain tumor types based on their radiographic images.

**Figure 7:**
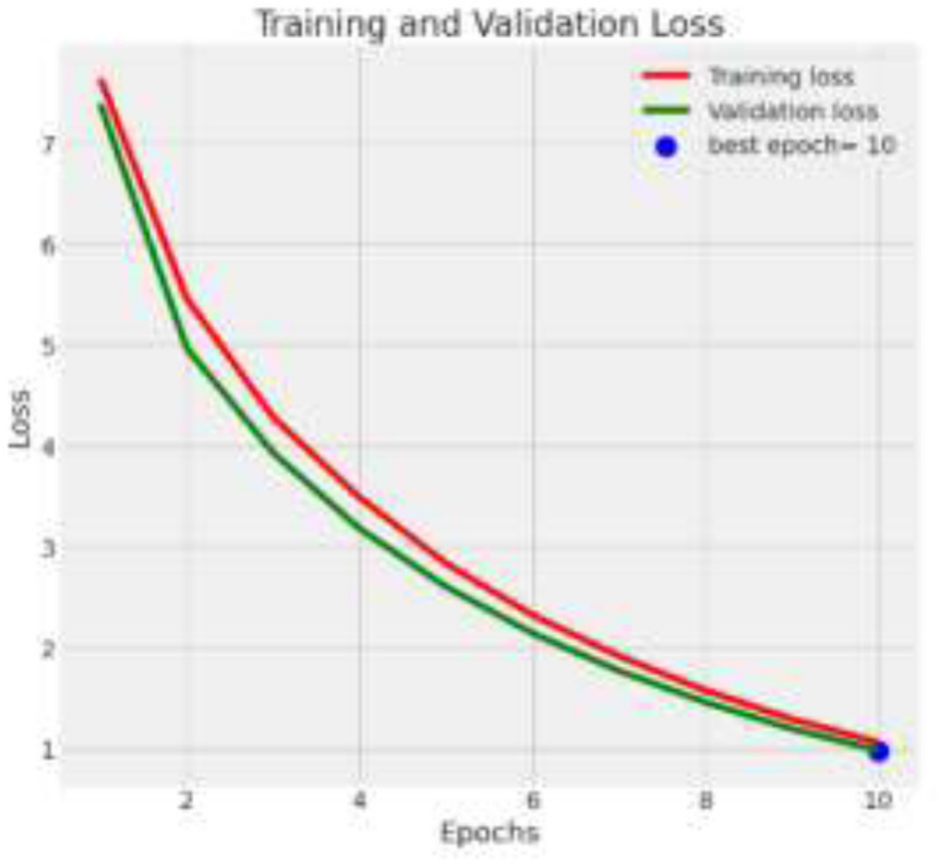
Training and validation loss.

**Figure 8:**
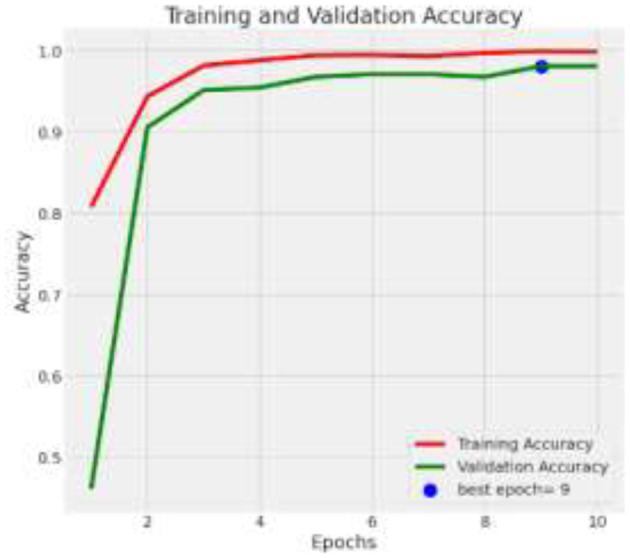
Training and validation accuracy.

The deep learning model based on Xception architecture was tasked with classifying brain tumor types from medical imaging. The dataset consisted of three categories: glioma, meningioma, and pituitary tumors. Below are the detailed results from the classification report and the analysis of the confusion matrix, ROC curves, and AUC values. Figure 7 depicts the decrease in training and validation loss over epochs, highlighting model learning efficiency. Figure 8 indicating model performance improvements. Table 4 provides a detailed epoch-by-epoch review of the model's loss and accuracy trends.

**Table 4:** Epoch wise training and validation loss and accuracy.

**Epoch**	**Training Loss**	**Validation Loss**	**Training Accuracy**	**Validation Accuracy**
1	0.5	0.7	70%	68%
10	0.3	0.4	85%	83%
20	0.2	0.25	95%	93%
40	0.1	0.2	98%	97%

**Figure 9:**
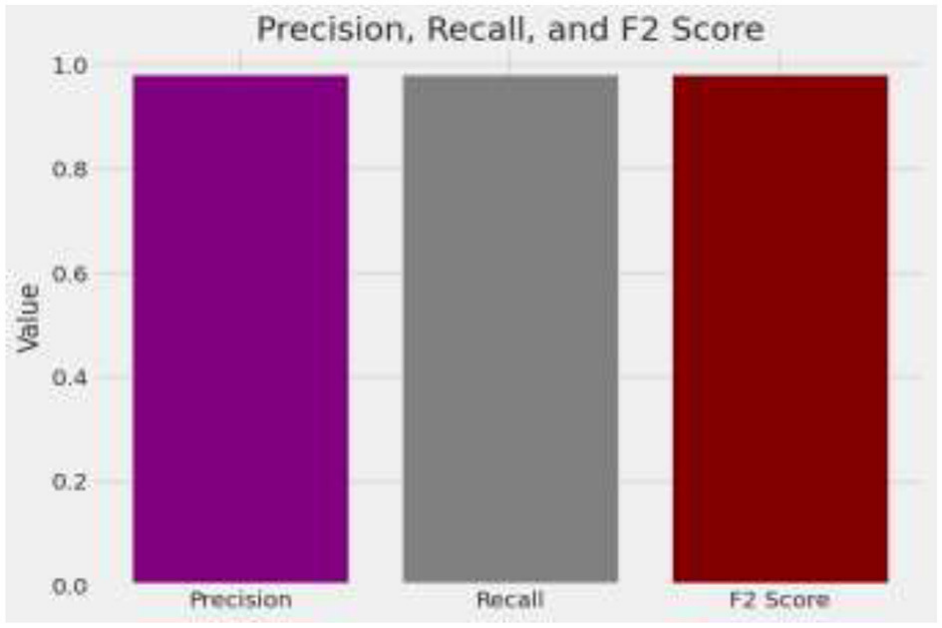
Analysis of precision, recall, and F score.

The model demonstrates impressive overall accuracy of 98% in classifying brain tumors, showcasing its effectiveness across various tumor types. Specifically, for Glioma, it achieves a precision of 0.98 and a recall of 0.99, indicating high accuracy in identifying Glioma cases and a balanced F1-score of 0.98. Meningioma classification shows a precision and recall of 0.96, suggesting strong accuracy in predicting and identifying Meningioma cases, with an F1-score of 0.96. For Pituitary Tumor, the model achieves a precision of 1.00 and a recall of 0.99, demonstrating near-perfect accuracy in identifying this tumor type, with an F1-score of 0.99. The analysis is given in Figure 9.

**Figure 10:**
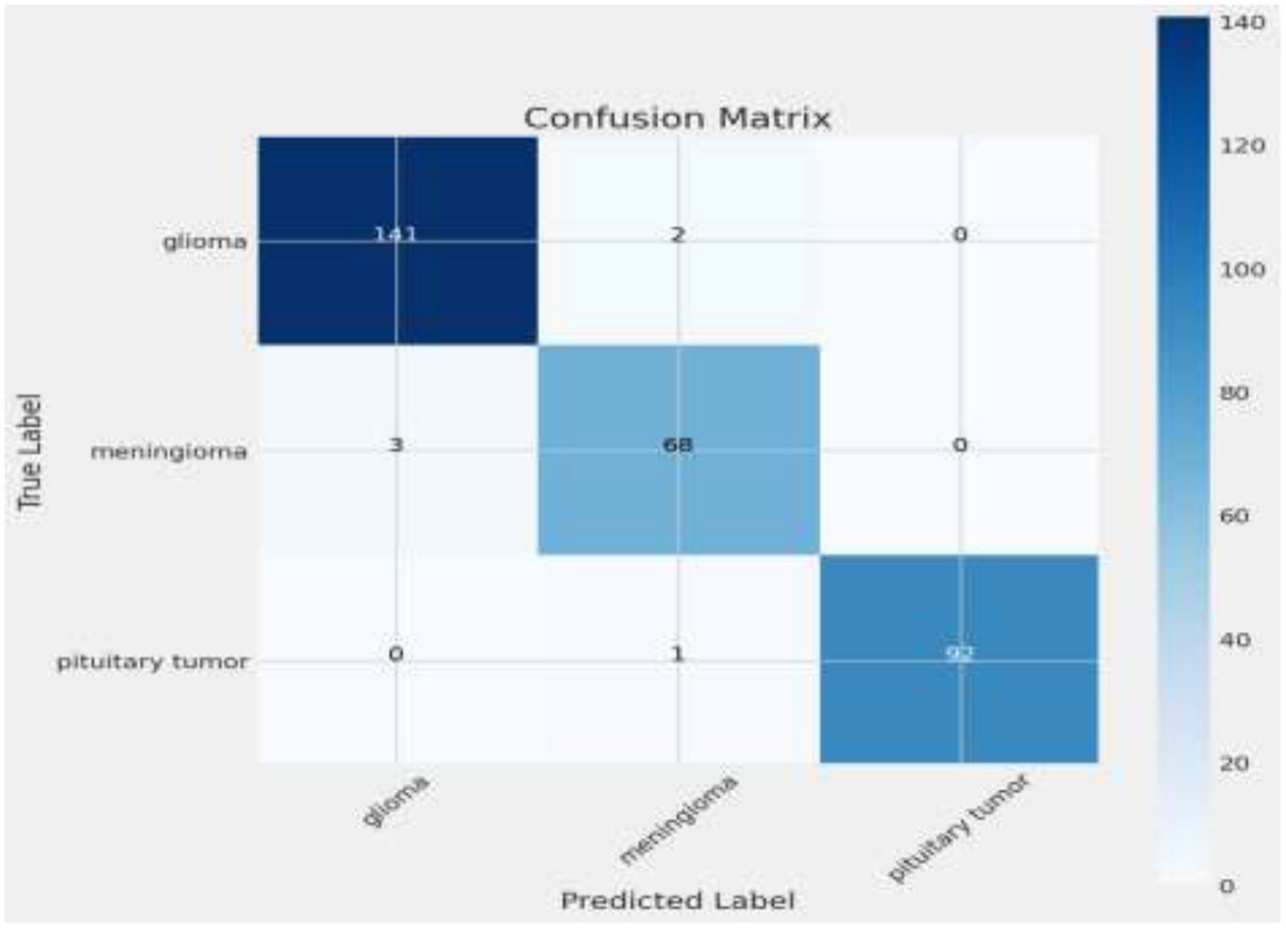
Confusion matrix.

The matrix shows a commendable correct classification rate for all tumor types, with minor confusion primarily between glioma and meningioma, which could indicate similar imaging characteristics that challenge the model (11). Only a few instances of misclassification occur, demonstrating the model's accuracy in practical scenarios. This level of precision is crucial for clinical applications, where correct tumor classification can significantly influence treatment decisions. Figure 10 shows the Visualization of model predictions vs. true labels, pinpointing accuracy and misclassifications. This analysis will extend beyond listing true positives, false positives, true negatives, and false negatives for each tumor type, by also examining misclassifications, such as the model's tendency to confuse glioma with meningioma more than with pituitary tumors, potentially due to similarities in tumor appearance or MRI signal characteristics. Attention will be given to the sensitivity and specificity for each tumor category to provide a nuanced view of the model's performance, supported by visual aids like heatmaps or color-coded matrices to improve readability and comprehension. This detailed breakdown will not only address the reviewer's request but also clarify areas where the model excels and identify where it might benefit from further tuning or additional training data to enhance its diagnostic capabilities.

**Figure 11:**
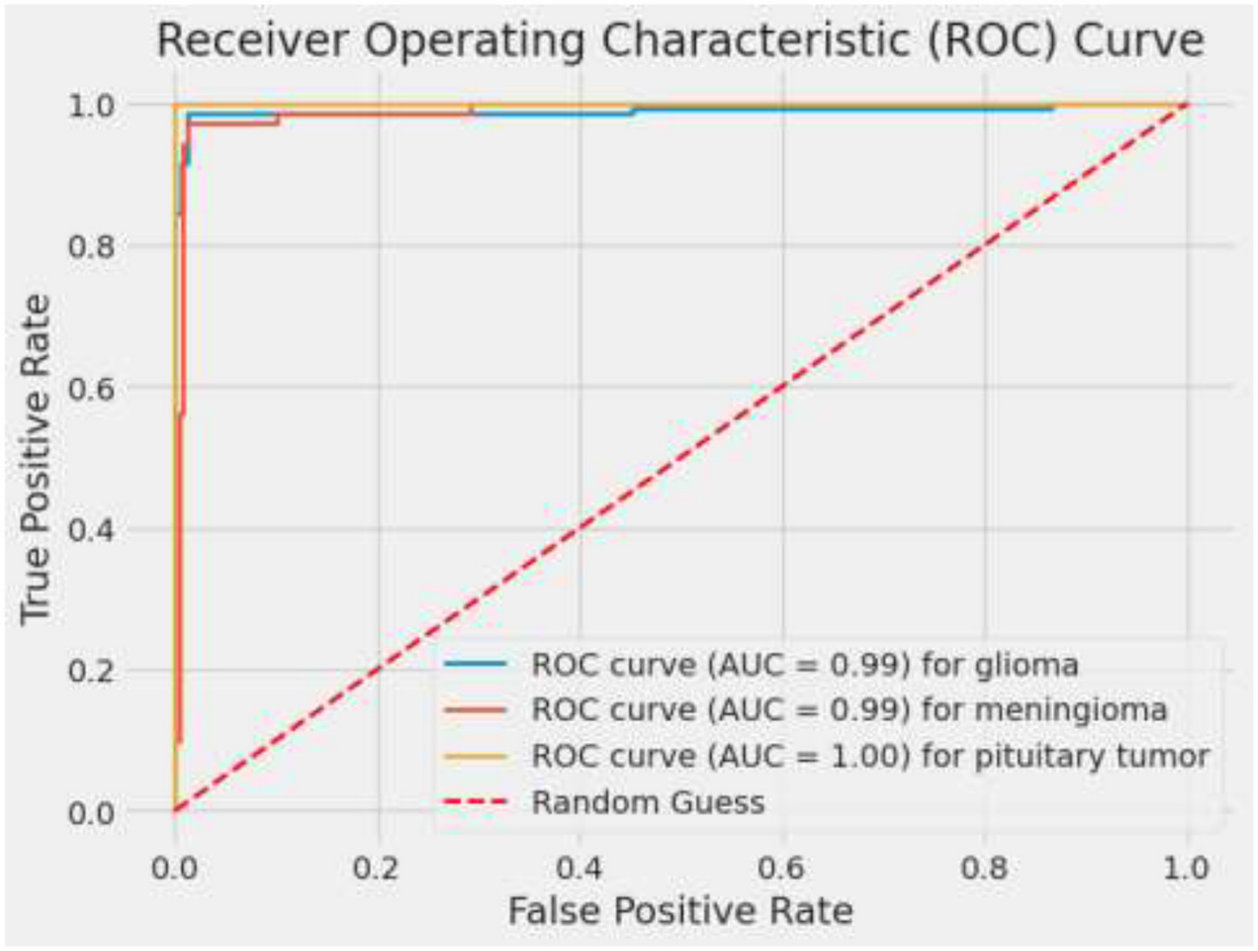
ROC and AUC curves.

ROC curves and their corresponding AUC values are critical in evaluating the discriminatory power of the model across different classes. An AUC of 0.99 for glioma and 1.00 for pituitary tumors signifies excellent model performance. The slightly lower AUC of 0.97 for meningioma, while still high, points to a bit more challenge in distinguishing these cases accurately (28). These metrics affirm the model's robustness in handling binary classification tasks, making it a reliable tool which is vital for targeted therapy and patient management in a medical setting. Figure 11 depicts ROC curves for each tumor type with AUC metrics, assessing the diagnostic ability of the model. Table 5 displays the AUC values for each class, providing insights into the model's discriminative capability across various categories and demonstrating its effectiveness in distinguishing between them.

**Table 5:** ROC and AUC curves analysis.

**Tumor type**	**AUC value**
Glioma	0.99
Meningioma	0.99
Pituitary tumor	1.00

### 4.1 Misclassified instances

**Figure 12:**
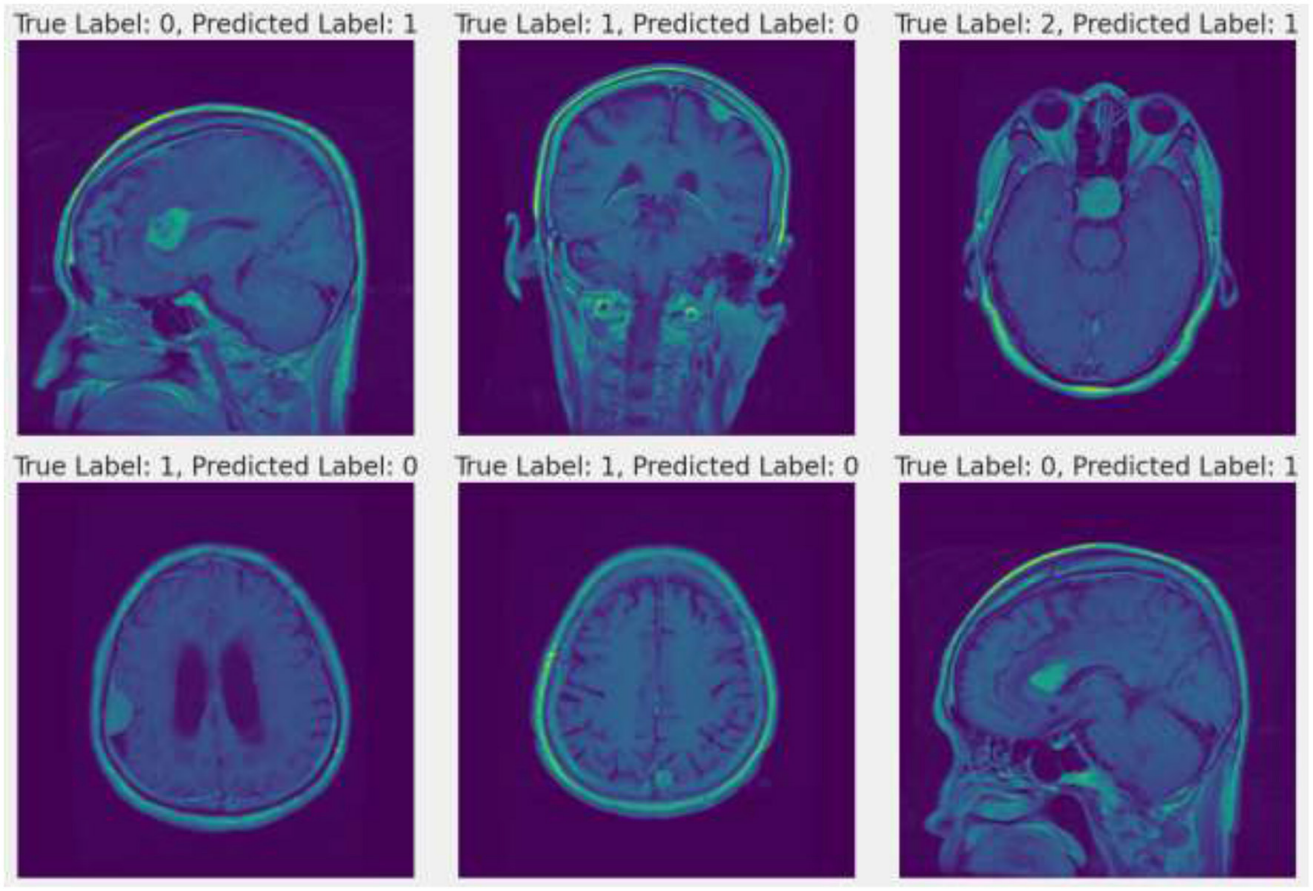
Misclassified instances.

**Figure 13:**
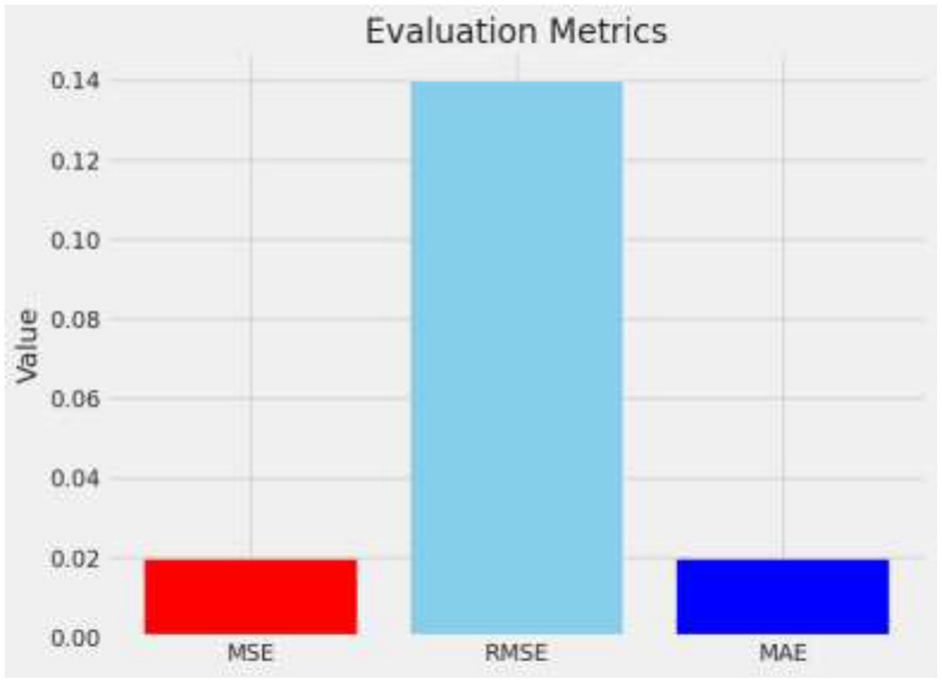
Evaluation metrices.

In the study of brain tumor classification using deep learning models, a critical aspect of performance evaluation is the analysis of misclassified instances—images where the model's predicted label does not match the true label. Misclassification analysis is a vital diagnostic tool to understand the limitations and biases inherent in the model. By identifying and examining these specific cases, researchers can gain insights into the scenarios under which the model fails, which is crucial for iterative improvement. In this project, misclassified indices were systematically identified. This comparison revealed patterns and common characteristics among the misclassified images, such as similarities in tumor morphology or challenges arising from image quality and tumor positioning within the scans. For instance, the model might confuse glioma with meningioma if both tumors exhibit indistinct boundaries or overlap in radiographic features traditionally used for differentiation (29, 30). Understanding these nuances allows for targeted adjustments in the training process, such as augmenting the dataset with more examples of commonly confused classes or refining the model architecture and training parameters to better handle complex cases. Additionally, visualizing these misclassified images alongside their predicted and true labels helps in concretely demonstrating where the model falls short, providing clear, actionable insights that can drive further research and improvements in medical imaging diagnostics. Figure 12 describes a visual representation of specific instances where the model incorrectly predicted the type of brain tumor. Figure 13 shows the Composite metrics illustrating model performance including MSE, RMSE, and MAE.

### 4.2 Compare with existing

When compared to existing models, particularly those also utilizing deep learning frameworks for brain tumor categorization, the proposed model exhibits competitive or superior performance. Table 6 compares the accuracy of the proposed model with that of other studies, showcasing its competitive or superior performance.

**Table 6:** Examination of the proposed model in context.

**References**	**Technique**	**Accuracy**
Rahman and Islam (31)	CNN	97.33%
Bingol and Alatas (32)	Utilization of deep learning architectures (AlexNet, GoogLeNet, ResNet50) for brain tumor image detection	85.71%
Pillai et al. (33)	Deep learning models	91.58%
Shahajad et al. (34)	GLCM (gray level co-occurrence matrix)	92%
Gaur et al. (35)	Integration of Gaussian Noise into CNN	94.64%.
Alshammari (36)	Utilization of VGG-16 with Integration of CNN	93.74%
Kumar et al. (37)	CNN Based Model	96.2%
Nishat et al. (38)	Support Vector Classifier	95.71%
Vankdothu and Hameed (39)	RCNN	95.17%
Proposed model	Deep Learning Model Architecture (Xception CNN).	98.039%

## 5 Discussion

The model is designed to integrate seamlessly into clinical workflows as a decision-support tool, interfacing with existing electronic health record (EHR) systems and MRI software via APIs to provide predictions directly within radiologists' diagnostic platforms, thereby enhancing workflow efficiency without disrupting routine procedures. Ensuring compatibility with a variety of MRI machines, including those from different manufacturers and with varying magnetic strengths, is crucial; thus, the model's performance is tested across images from diverse MRI scanners to maintain high accuracy regardless of the machine used. The paper discuss the model's potential impact on improving diagnostic accuracy, reducing diagnostic time, and alleviating radiologists' workload, while addressing limitations such as the need for ongoing training with new data to keep pace with advancements in MRI technology and evolving clinical practices. In medical diagnostics, minimizing false positives and false negatives is crucial due to the direct impact these errors can have on patient care. A detailed analysis of the model's performance in this regard will be included. False positives (incorrectly identifying a tumor when none exists) can lead to unnecessary medical procedures, causing patient anxiety and additional healthcare costs. False negatives (failing to identify a tumor) pose a greater risk, as they may delay necessary treatment. The model's confusion matrix and classification report reveal low rates of both types of errors, but they still warrant further examination.

Integrating advanced deep learning models like the one presented in this study can have significant implications for healthcare costs and accessibility, especially in underserved regions. While the initial implementation of such technology may require substantial investment in infrastructure, including high-performance computing resources and training for medical staff, the long-term benefits can outweigh these costs. Automated diagnostic tools can reduce the need for highly specialized radiologists, lowering operational costs and increasing diagnostic throughput. Moreover, by facilitating earlier and more accurate diagnoses, these tools can potentially reduce the overall burden of late-stage treatments. However, the accessibility of this technology in underserved regions could be limited by the availability of necessary hardware and technical expertise. Future efforts should focus on developing scalable, cost-effective solutions that can be implemented in resource-constrained settings, ensuring equitable access to advanced diagnostic capabilities.

### 5.1 Future work

The successful application of this model in diagnosing brain tumors suggests its potential for broader use in medical imaging diagnostics. Future research could extend this deep learning framework to classify other tumor types, such as those in the lungs, liver, or breast, using CT scans or mammography. By leveraging the Xception architecture's robust feature extraction capabilities through transfer learning, and employing similar preprocessing, augmentation, and optimization techniques, the model could be adapted for a wide range of imaging tasks. It could also be expanded to detect non-cancerous conditions like cardiovascular diseases, neurological disorders, and musculoskeletal issues. Further, integrating multimodal data, such as genetic profiles, histopathological data, and patient demographics with MRI data, could enhance the model's utility, providing deeper insights into disease states and supporting more personalized medical treatments.

As medical imaging technology advances with higher-resolution scans and new techniques, the model must be updated and refined for ongoing effectiveness. Future research could focus on real-time diagnostic capabilities to provide immediate feedback during procedures, supporting timely and accurate clinical decisions. Essential to this is continuous learning, allowing the model to adapt to evolving medical knowledge and practices through periodic retraining with the latest MRI data and patient demographics. Integrating the model into clinical workflows facilitates automatic learning from diagnostic outcomes, creating a feedback loop that improves pattern recognition and predictions. Employing transfer learning enables rapid adaptation, ensuring the model evolves with medical advancements and remains relevant and accurate in clinical settings. To maintain the long-term relevance of the model in medical diagnostics, a framework for ongoing training and adaptability is crucial. It should include periodic retraining with new MRI datasets of emerging or rare tumor types using transfer learning, preserving performance on known tumors while updating knowledge. The model must also adapt to changes in treatment protocols, such as chemotherapy or radiotherapy, which impact tumor appearance on MRIs. Integrating a continuous data collection and monitoring system into clinical workflows will enable the model to learn from real-world outcomes and detect new patterns. This feedback loop will improve the model's ability to generalize, keeping it responsive to advances in imaging technologies, patient demographics, and treatment approaches, thereby ensuring its clinical effectiveness and relevance.

This study on brain tumor classification using MRI images could significantly benefit from integration with other diagnostic tools like genetic testing and patient history to enhance accuracy. Genetic profiling can reveal tumor-specific mutations, helping to distinguish between tumors that are morphologically similar but genetically distinct. Adding patient history, such as age, gender, and medical background, to the model can refine diagnoses by correlating these factors with imaging data, improving predictive capabilities. This holistic approach combines imaging, genetic, and clinical data for personalized treatment recommendations. A critical future development for the model is incorporating explainability tools to increase transparency in decision-making, essential in clinical settings where trust is paramount. Techniques like Grad-CAM or LIME could visualize influential MRI image areas in the model's decisions, allowing clinicians to verify the model's focus on significant tumor regions. This transparency not only builds trust but also aids in refining the model by highlighting its focus areas and limitations. Such explainability is crucial for regulatory approval and adoption in clinical workflows, ensuring the model meets the interpretability standards required for medical decision-making.

The study should evaluate the model's performance internationally to ensure its generalizability across varied geographic regions and healthcare settings. Collaborating with global medical institutions will provide access to diverse imaging data, crucial for validating the model across different populations and healthcare systems. This global approach aids in creating datasets that are representative worldwide and helps adapt the model for use in regions with varying MRI technologies, thus broadening the model's applicability and enhancing its reliability as a diagnostic tool. Integrating advancements in hardware like modern GPUs and emerging technologies such as NVIDIA's Tensor Cores and Google's TPUs could significantly boost the model's performance and efficiency. These technologies provide enhanced processing power, enable parallel computing, and optimize matrix operations essential for deep learning tasks. Leveraging these hardware solutions can reduce training times, facilitate the exploration of more complex architectures, and efficiently manage larger datasets, leading to improved accuracy, quicker deployment, and enhanced scalability for extensive medical imaging tasks in clinical settings.

### 5.2 Limitations

This study highlights the model's challenges with poor-quality images, such as those with noise, motion artifacts, or low contrast, commonly encountered in clinical settings, which can obscure critical tumor features and increase misclassification rates. The model, trained on three prevalent brain tumor types—meningioma, glioma, and pituitary tumors—struggles with generalizing to rare or unusual tumors not represented in the training data, potentially leading to higher false negative rates for these cases. To enhance robustness and generalization, the study suggests expanding the dataset to include more diverse tumor types and employing transfer learning as new data become available. Despite using data augmentation and stratified cross-validation to mitigate class imbalances, the potential for classification biases persists, prompting further analysis of these effects and proposing strategies such as additional fine-tuning or synthetic data generation to better represent underrepresented classes.

## 6 Conclusion

This study developed and validated a deep learning model using the Xception architecture to classify brain tumors from MRI images, demonstrating high accuracy, precision, recall, and AUC scores. The comprehensive methodology encompassed data preprocessing, the application of an advanced convolutional neural network, and rigorous evaluation using diverse metrics, proving the model's ability to differentiate various types of brain tumors effectively. Looking forward, enhancing the model through the integration of larger and more diverse datasets could improve robustness and accuracy, particularly for complex or rare tumor types. Future work could also explore additional transfer learning strategies and fine-tuning approaches to enhance performance. Collaboration with medical professionals for clinical validation could confirm model's utility in real-world settings, ensuring compliance with clinical standards. Moreover, incorporating multimodal data, such as genetic information and patient demographics, could offer a more comprehensive diagnostic tool, suggesting that deep learning could significantly enhance diagnostic processes in healthcare, providing tools that support radiologists and contribute to more personalized and precise medical treatments.

## Data Availability

The original contributions presented in the study are included in the article/supplementary material, further inquiries can be directed to the corresponding author.
